# Posttraumatic Stress Disorder and Pregnancy Outcomes

**DOI:** 10.1097/og9.0000000000000060

**Published:** 2025-02-06

**Authors:** Monica A. Lutgendorf, Radhika Tyagi, Sarah Edwards, Shad Deering, Sorana Raiciulescu, Robert B. Walton, Jason A. Pates, Peter G. Napolitano, Nicholas Ieronimakis

**Affiliations:** Department of Gynecologic Surgery and Obstetrics and the Department of Preventive Medicine and Biostatistics, Uniformed Services University of the Health Sciences, Bethesda, Maryland; the Division of Maternal Fetal Medicine and the Department of Clinical Investigation, Madigan Army Medical Center, Tacoma, and the Department of Obstetrics and Gynecology, University of Washington, Seattle, Washington; the Department of Obstetrics and Gynecology, Baylor College of Medicine, San Antonio, Texas; and the Department of Obstetrics and Gynecology, Naval Medical Center Camp Lejeune, Camp Lejeune, North Carolina.

## Abstract

**OBJECTIVE:**

To evaluate the prevalence and effect of posttraumatic stress disorder (PTSD) on pregnancy outcomes among individuals with military health care.

**METHODS:**

This was a prospective cohort study of pregnant individuals receiving care at a military tertiary care center from 2014 to 2018. Participants were screened for PTSD with the PTSD Checklist for civilians and for military service members, as well as for traumatic experiences, combat exposure, risk drinking, and depression. Pregnancy outcomes included preterm birth, fetal growth restriction, preeclampsia, gestational diabetes mellitus, hypertensive disorders of pregnancy, outpatient visits, and neonatal intensive care unit admissions.

**RESULTS:**

Among 1,467 pregnant individuals, 1,145 were retained: 298 (26.0%) military service members and 847 (73.9%) civilian family members. A total of 108 individuals screened positive for PTSD: 21 (7.0%) military service members and 87 (10.3%) civilians. Individuals who screened positive for PTSD had a 17-fold increased likelihood for depression (50.9% vs 5.5%, odds ratio [OR] 17.84, 95% CI, 11.24–28.33), a fivefold increased likelihood for risk of alcohol use (14.8% vs 3.0%, OR 5.64, 95% CI, 2.97–10.69), and a greater number of outpatient visits (median 18 vs 22.5, *P*=.007). Pregnancy complications were not different between military service members and civilians or those who screened positive for PTSD.

**CONCLUSION:**

Screening results positive for PTSD are associated with positive screen results for depression and risk of alcohol use during pregnancy, with no differences in outcomes. Although we identified no differences in pregnancy outcomes based on PTSD screening results, comorbidities of traumatic stress such as depression and alcohol use disorder may have long-term consequences on maternal and fetal health. Further research is warranted to understand the effect of behavioral health disorders on pregnancy.

## Abstract 2

Posttraumatic stress disorder affects 7–10% of pregnancies and is associated with an increased risk of individuals screening positive for depression and at-risk alcohol use.

Posttraumatic stress disorder (PTSD) is characterized by experience of a traumatic event, symptoms of recurrent, intrusive recollections, symptoms of avoidance, negative alterations of mood and cognition, numbing, and hyperarousal.^1^ Posttraumatic stress disorder can arise from traumatic exposures, with an estimated lifetime prevalence of 9.7–12.3% in population-based surveys,^2,3^ rates of 25–50% among women exposed to abuse or assault, and increased risks in women.^3,4^ Posttraumatic stress disorder is more prevalent among female veterans compared with female civilians, male veterans, and male civilians.^5^ Female veterans reported a variety of traumatic events, including childhood abuse, interpersonal violence, combat or exposure to a war zone, severe illness or injury, and natural disasters.^5^

Studies investigating PTSD in veterans reveal higher rates among women, with lifetime rates of 13.4% compared with civilian woman (8.0%), male veterans (7.7%), and civilians (3.4%).^5^ Past-year rates of PTSD in female veterans were 11.7% compared with 6.0% for female civilians, 6.7% for male veterans, and 2.6% for male civilians.^5^ Female service members with PTSD are more likely to have comorbid adjustment, depressive, anxiety, eating, and personality disorders and less likely to have comorbid alcohol or substance use disorders, insomnia, and traumatic brain injury compared with male service members.^6^ The type of PTSD may relate to types and frequency of trauma in female veterans,^7^ with studies showing moderately higher rates of PTSD attributed to military service, including overseas operations and combat.^8–13^ Health effects of PTSD can lead to increased use of health care services; risk for depression, suicide, and alcohol and substance use; and other effects, including pregnancy complications.^6,8,14–19^

The prevalence of PTSD in pregnancy is 2.3–8.1%.^19,20^ Posttraumatic stress disorder may be associated with higher complications such as preterm birth, alcohol or substance use, and poor prenatal care,^14,16–18,21^ particularly in individuals with lifetime trauma exposures.^22^ In a recent retrospective cohort study of PTSD in pregnant military servicewomen, the prevalence of PTSD was 1.7%, and mental health comorbidities were common: depression in 60.9%, adjustment disorder in 43.4%, and anxiety disorder in 39.3%.^15^ Retrospective studies have demonstrated increased risks for gestational diabetes mellitus, hypertension, and preterm birth in veterans diagnosed with PTSD within a year of delivery.^16^

Perinatal depression is common, affecting 6.0–16.6% in the general population.^23,24^ Among individuals with military health care, perinatal depression may be more common: 6.5–24% in servicewomen and 4.6–50.7% in military spouses.^25^ Mental health and alcohol use disorders are common with PTSD^14,15^ and are important comorbidities in pregnancy. Although studies have assessed outcomes of PTSD^17,18,20^ and depression^23,24^ in the perinatal period, prospective outcomes between military and civilian populations remain understudied,^19,25^ and military populations may have higher rates of PTSD and adverse outcomes. Women make up 17.1% of military service members^26^ and are the fastest growing segment of the veteran population, with more than 2 million female veterans in the United States.^27^ Furthermore, the Military Health System covers 89,000 annual births, with 75,400 of these births in the purchased care (civilian) market; thus, military service members and their family may be seen in civilian practices.^28^ We hypothesized that rates of PTSD and adverse outcomes would be higher in military-affiliated pregnant populations. The objective of this study was to determine the prevalence of positive PTSD screen results and the effect on pregnancy outcomes (preterm delivery, perinatal depression, alcohol use, fetal growth restriction, preeclampsia, gestational diabetes, neonatal intensive care unit [NICU] admission and length of stay, and number of outpatient encounters) among individuals with military health care (military service members and civilian family members).

## METHODS

This study was approved by the IRB at Madigan Army Medical Center and conforms with the U.S. Federal Policy for the Protection of Human Subjects. This was a prospective cohort study of patients delivering at a tertiary academic facility (military treatment facility), Madigan Army Medical Center in Tacoma, Washington, from January 2014 to January 2018. Eligible pregnant individuals included military service members and family members. Inclusion criteria included age 18 years or older with first prenatal visit at 16 weeks of gestation or earlier. Individuals were excluded if they declined, did not speak English, or were at more than 16 weeks of gestation. After providing consent, participants were screened for PTSD, traumatic event exposure, combat exposure (if military), depression, and at-risk alcohol use at presentation for prenatal care. Screening instruments were administered to participants at their first prenatal visit at or before 16 weeks of gestation, and pregnancy outcome data were collected from the electronic medical record by study nurses.

Screening for PTSD was completed with the PTSD Checklist (PCL) military (PCL-M) and civilian (PCL-C).^29,30^ This study was conducted before the PCL update for the *Diagnostic and Statistical Manual of Mental Disorders* (Fifth Edition). The PCL is a 17-item self-report measure of the 17 *Diagnostic and Statistical Manual of Mental Disorders* (Fourth Edition) symptoms of PTSD. This checklist can be used for screening and diagnosing PTSD and monitoring symptoms during treatment.^29^ The checklist has been validated for use in military and civilian populations.^30,31^ A score of 38 or higher on either PCL determined positive PTSD screen results.^32–35^

Instruments related to trauma and comorbidities included Trauma History Screen, Combat Exposure Scale, Edinburgh Postnatal Depression Scale, and T-ACE screen for at-risk drinking. The Trauma History Screen identifies exposures to events associated with posttraumatic distress.^36,37^ The Combat Exposure Scale is a seven-item assessment of wartime stressors experienced by combatants.^38,39^ A Combat Exposure Scale score of 0–8 is light exposure, 9–16 is light to moderate exposure, 17–24 is moderate exposure, 25–32 is moderate to heavy exposure, and 33–41 is heavy exposure to combat.^38,39^ The Edinburgh Postnatal Depression Scale was used to screen for depression,^40^ with a score of 12 or higher considered positive.^41–43^ Finally, for the T-ACE, a score of 2 or higher was positive for at-risk drinking (consumption of 1 ounce or more of alcohol per day).^44^ All participants completed the PCL-C or PCL-M, Edinburgh Postnatal Depression Scale, Trauma History Screen, and T-ACE; individuals with military experience completed the PCL-M and the Combat Exposure Scale.

The primary exposure was a positive PTSD screen result. Primary outcomes were preterm delivery (before 37 weeks of gestation), perinatal depression (positive Edinburgh Postnatal Depression Scale), and positive screen result on T-ACE (score 2 or higher) for at-risk drinking. Secondary outcomes included fetal growth restriction, preeclampsia, gestational diabetes, NICU admission, length of NICU admission, and number of outpatient encounters. Data were collected from surveys and chart review by trained study nurses, including pregnancy and delivery records. The sample size was calculated using α of 5% and power of 80%. For preterm delivery, with a baseline risk of 8%, 215 individuals who screened positive for PTSD were needed to detect an increase to 16%. For perinatal depression, with a baseline risk of 19%, 239 individuals screening positive for PTSD were needed to detect an increase to 30%. Using an interim analysis of 494 individuals with a positive PTSD screen rate of 11.6%, we calculated that a sample size of 2,060 individuals was needed to enroll 239 with positive PTSD screen results. Race was included as a variable of interest because mood disorders have been found to differ on the basis of race with high psychiatric symptom burden,^22^ which may be attributable to social factors or stressors, including systemic oppression or racism.

Data were summarized with means and SDs or frequency and percentages. Unadjusted comparisons between military and civilian participants and those who screened positive for PTSD were completed with a χ^2^ test for categorical variables. Continuous variables were assessed with a Mann–Whitney *U* test or an independent-samples *t* test, depending on data distribution. Multivariate logistic regression models were constructed for dichotomous outcomes. Models were adjusted for relevant control variables, including history of depression, tobacco use, education, marital status, parity, body mass index (BMI, calculated as weight in kilograms divided by height in meters squared) before pregnancy, and the main effects of military or civilian status and PTSD status, along with an interaction of the two. Subsequent between-group and within-group pairwise comparisons were run. Logistic models are summarized with odds ratios (ORs) and 95% CIs. Statistical analyses were completed with IBM SPSS 24. Differences are considered significant if *P*<.05. There were no imputation for missing data and no correction for multiple comparisons.

## RESULTS

**Fig. 1.**
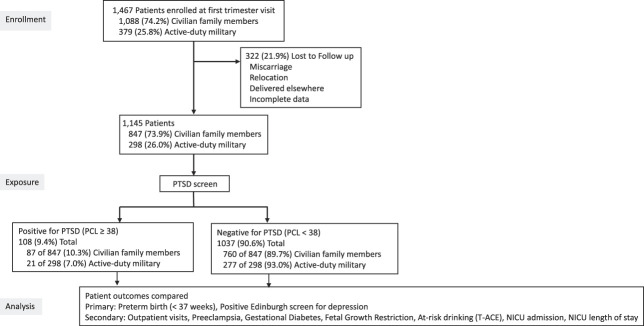
STROBE (Strengthening the Reporting of Observational Studies in Epidemiology) enrollment flowchart depicting the sequence of this study from enrollment of pregnant civilian and military participants. PTSD, posttraumatic stress disorder; PCL, PTSD Checklist; NICU, neonatal intensive care unit.

From January 2014 to December 2018, 1,467 participants enrolled. Of these, 21.9% (n=322) were excluded because of miscarriage, relocation, or missing data (Fig. 1). Among 1,145 participants analyzed, 9.4% (n=108) screened positive for PTSD. There were significant differences in characteristics between individuals who screened positive and those who screened negative for PTSD. Those who screened positive were younger, less likely to have a college degree, and more likely to be divorced or separated (Table 1). There was a small significant difference in gestational age at delivery and BMI. There were no differences in rates of PTSD between military (7.0%) and civilian (10.3%, *P*=.101) participants (Table 1). A greater proportion of participants who screened PTSD positive compared with those who screened negative at their initial obstetrician appointment (at or before 16 weeks of gestation) reported having previously experienced anxiety, depression, or PTSD with prior or ongoing treatment with counseling, medications, and prior hospitalization (Appendix 1, available online at http://links.lww.com/AOG/D986).

*Lutgendorf. PTSD and Pregnancy Outcomes. O&G Open 2025*.

**Table 1.** Demographic and Clinical Characteristics of Pregnant Participants (Civilian and Military Combined) Who Screened Negative and Those Who Screened Positive for Posttraumatic Stress Disorder

Characteristic	PTSD Screen Negative (n=1,037)*	PTSD Screen Positive (n=108)†	*P* ‡
Civilian family member	760 (89.7)	87 (10.3)	.101
Active-duty military	277 (93.0)	21 (7.0)	
Age (y)	27.1±5.2	26.0±5.3	.034
BMI (kg/m^2^)			
Before pregnancy	26.6±6.0	27.5±5.8	.108
After delivery	31.8±5.9	33.0±5.8	.036
Gestational age at delivery (wk)	39.0±1.7	38.6±2.0	.031
Gravidity§	2 (9)	2 (11)	.072
Parity§	1 (8)	1 (4)	.184
Birth weight (g)	3,354.1±532.2	3,372.5±565.3	.734
Twin pregnancy	16 (1.5)	1 (0.9)	.614
Mode of delivery			
Vaginal (no forceps or vacuum)	797 (76.9)	80 (74.1)	.708
Vaginal with forceps	11 (1.1)	3 (2.8)	
Vaginal with vacuum	14 (1.4)	2 (1.9)	
Primary cesarean	121 (11.7)	12 (11.1)	
Repeat cesarean	92 (8.9)	11 (10.2)	
Maternal race and ethnicity			
African American	106 (10.2)	12 (11.1)	.692
American Indian/Alaska Native	9 (0.9)	2 (1.9)	
Asian or Pacific Islander	100 (9.6)	5 (4.6)	
Hispanic	134 (12.9)	14 (13.0)	
Multiracial	92 (8.9)	10 (9.3)	
White	586 (56.5)	64 (59.3)	
None of the above	10 (1)	1 (0.9)	
Highest level of education			
Some high school	20 (1.9)	8 (7.4)	<.001
High school	187 (18.0)	29 (26.9)	
Some college	346 (33.4)	46 (42.6)	
2-y degree	117 (11.3)	12 (11.1)	
4-y degree	252 (24.3)	9 (8.3)	
Graduate degree	115 (11.1)	4 (3.7)	
Marital status			
Married	954 (92.1)	94 (87.0)	.030
Single never married	62 (6.0)	7 (6.5)	
Separated	6 (0.6)	2 (1.9)	
Divorced	12 (1.2)	5 (4.6)	
Widowed	2 (0.2)	0 (0.0)	

PTSD, posttraumatic stress disorder; BMI, body mass index.

Data are n (%) or mean±SD unless otherwise specified.

* Posttraumatic Stress Disorder Checklist score lower than 38.

† Posttraumatic Stress Disorder Checklist score 38 or higher.

‡ *P* values are from χ^2^ or Student *t* test as appropriate.

§ Median (range).

Compared with those who screened negative, participants who screened positive for PTSD were 17 times more likely to screen positive for depression (OR 17.84, 95% CI, 11.24–28.33) and five times more likely to screen positive for at-risk alcohol use (OR 5.64, 95% CI, 2.97–10.69) (Table 2). Participants who screened positive for PTSD were significantly more likely to report tobacco use (OR 4.24, 95% CI, 2.04–8.81) and to have four more outpatient visits (median 22.5) compared with participants who screened negative for PTSD (median 18). There were no significant differences between individuals who screened PTSD positive and those who screened negative for pregnancy outcomes, including preterm delivery, growth restriction, preeclampsia, gestational diabetes, gestational hypertension, NICU admission, or NICU length of stay (Table 2).

**Table 2.** Depression and Alcohol Screening Results and Pregnancy Outcomes for Pregnant Participants (Civilian and Military Combined) Who Screened Negative and Those Who Screened Positive for Posttraumatic Stress Disorder*

Outcome	PTSD Screen Result		*P* †	OR (95% CI) (Ref: PTSD Screen Negative)
	Negative (n=1,037)	Positive (n=108)		
Depression screen positive (EDPS score 12 or higher)	57 (5.5)	55 (50.9)	<.001	17.84 (11.24–28.33)
Alcohol screen positive (T-ACE score 2 or higher)	31 (3.0)	16 (14.8)	<.001	5.64 (2.97–10.69)
Tobacco use	27 (2.6)	11 (10.2)	<.001	4.24 (2.04–8.81)
Premature birth (before 37 wk)	92 (8.9)	12 (11.1)	.441	1.28 (0.68–2.43)
Fetal growth restriction	18 (1.7)	1 (0.9)	.531	0.54 (0.07–4.00)
Preeclampsia	15 (1.4)	3 (2.8)	.290	1.95 (0.56–6.83)
Gestational diabetes	66 (6.4)	11 (10.2)	.131	1.67 (0.85–3.27)
Gestational hypertension	77 (7.4)	13 (12.0)	.090	1.71 (0.91–3.19)
Outpatient visits (n)	18±12‡	22.5±21‡	.007	—
Obstetrician clinic visits (n)	9±3‡	10±4‡	.272	—
NICU admission	105 (10.1)	16 (14.8)	.131	1.54 (0.88–2.72)
NICU length of stay (d)	6±9‡	10±6‡	.055	—

PTSD, posttraumatic stress disorder; OR, odds ratio; Ref, referent; EDPS, Edinburgh Postnatal Depression Scale; NICU, neonatal intensive care unit.

Data are n (%) or mean±SD unless otherwise specified.

* Within respective groups, n (%) reflects participants who screened positive for EDPS or T-ACE or experienced pregnancy complications or NICU admission. The mean±SD of outpatient visits during pregnancy and NICU length of stay in days are compared between groups.

† *P* values are from χ^2^ or Mann–Whitney *U* test as appropriate.

‡ Summarized with median (interquartile range).

**Fig. 2.**
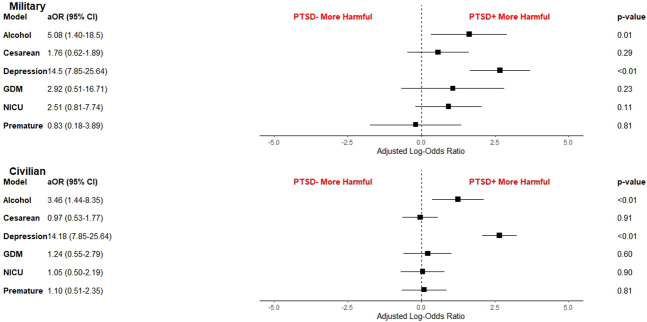
Adjusted odds ratios (aOR) of outcomes. GDM, gestational diabetes mellitus; NICU, neonatal intensive care unit; PTSD, posttraumatic stress disorder.

After adjustment for control variables, the association between positive PTSD screen results and depression remained, with an adjusted OR of 14.5 (95% CI, 7.85–25.64) for military service members and 14.1 (95% CI, 7.85–25.64) for civilian family members (Fig. 2). The association between positive PTSD screen results and at-risk alcohol use also remained, with an adjusted OR of 5.08 (95% CI, 1.4–18.5) for military service members and 3.46 (95% CI, 1.44–8.35) for civilian family members (Fig. 2).

*Lutgendorf. PTSD and Pregnancy Outcomes. O&G Open 2025*.

Among participants who screened positive for PTSD, there was no difference between military service members and civilian family members for depression, at-risk alcohol use, and most pregnancy and fetal outcomes (Table 3). Military service members with PTSD had almost twice the number of outpatient visits compared with civilian family members who screened positive for PTSD (Table 3). When analyzed in respective cohorts, both civilian family members and military service members who screened positive for PTSD had higher rates of depression and at-risk alcohol use and more outpatient visits (Appendix 2, available online at http://links.lww.com/AOG/D986).

**Table 3.** Depression and Alcohol Screening Results and Pregnancy Outcomes for Pregnant Civilian and Military Participants Who Screened Positive for Posttraumatic Stress Disorder*

Outcome	Civilian Family Members (n=87)	Military Service Members (n=21)	*P* †
Depression screen positive (EDPS score 12 or higher)	43 (49.4)	12 (57.1)	.525
Alcohol screen positive (T-ACE score 2 or higher)	11 (12.6)	5 (23.8)	.196
Tobacco use	9 (10.3)	2 (9.5)	.911
Premature birth (before 37 wk)	10 (11.5)	2 (9.5)	.796
Fetal growth restriction	1 (1.1)	0 (0.0)	.622
Preeclampsia	2 (2.3)	1 (2.3)	.538
Gestational diabetes	9 (10.3)	2 (9.5)	.911
Gestational hypertension	1 (1.1)	12 (57.1)	.254
Cesarean delivery	17 (19.5)	6 (28.6)	.365
Outpatient visits (n)	18.3±0.9	36.9±4	<.001
NICU admission	11 (12.6)	5 (23.8)	.196
NICU length of stay (d)	13.9±12.5	7.4±2.8	.145

EDPS, Edinburgh Postnatal Depression Scale; NICU, neonatal intensive care unit.

Data are n (%) or mean±SD unless otherwise specified.

* Within respective groups, n (%) reflects participants who screened positive for EDPS or T-ACE instruments or who experienced pregnancy complications and NICU admission. The mean±SD of outpatient visits during pregnancy and NICU length of stay in days are compared between groups.

† *P* values are from χ^2^ or Mann–Whitney *U* test as appropriate.

Among all participants, a greater proportion of individuals who screened positive for PTSD reported experiencing trauma and at a greater frequency than those who screened negative (Table 4). Among those who screened positive for PTSD, military service members reported a significantly higher frequency of witnessing a bad accident, seeing something horrible during military service, or experiencing physical or sexual assault as an adult compared with civilians (Table 5). The frequency of the traumatic experiences was not significantly different between military and civilian participants who screened positive for PTSD (Table 5). For military participants, reported levels of combat exposure were also greater for those who screened positive for PTSD relative to those who screened negative (Table 6).

**Table 4.** Proportion (Relative to the Total Number of Posttraumatic Stress Disorder Negative and Positive Participants) and Average Frequency That Pregnant Participants (Civilian and Military Combined) Reported Experiencing Traumatic Events on the Trauma History Screen

Trauma History Screen Question	Proportion			Frequency			
	PTSD Screen Negative (n=1,037)	PTSD Screen Positive (n=108)	*P* *	PTSD Screen Negative (n=1,037)	PTSD Screen Positive (n=108)	*P* *
Witnessing a really bad car, boat, train, or airplane accident	173 (16.7)	33 (30.6)	<.001	1.4±0.9	1.5±0.8	.354
Witnessing a really bad accident at work or home	43 (4.1)	10 (9.3)	.016	3.8±15.1	11.7±30.7	.079
Witnessing a hurricane, flood, earthquake, tornado, or fire	177 (17.1)	30 (27.8)	.006	5.1±16.3	12.1±30.6	.350
Hit or kicked hard enough to injure as a child	62 (6.0)	27 (25.0)	<.001	26.7±42.7	36.3±46.8	.107
Hit or kicked hard enough to injure as an adult	58 (5.6)	14 (13.1)	.002	14.1±32.2	24.9±42.3	.133
Forced or made to have sexual contact as a child	96 (9.3)	40 (37.7)	<.001	14.8±33.7	79.4±43.8	**.003**
Forced or made to have sexual contact as an adult	66 (6.6)	26 (24.3)	<.001	7.7±24.0	5.8±19.4	**.013**
Attack with a gun, knife, or weapon	30 (2.9)	12 (11.2)	<.001	4.7±18.2	1.6±1.0	.835
During military service, seeing something horrible/being badly scared	44 (4.2)	15 (14.0)	<.001	4.2±15.4	1.9±0.9	.280
Sudden death of a close family member or friend	373 (36.0)	62 (57.4)	<.001	2.1±5.2	3.4±12.7	.817
Seeing someone die suddenly or get badly hurt or killed	128 (12.3)	32 (29.9)	<.001	7.1±22.6	4.9±18.1	.602
Some other event that made you feel very scared, helpless, or horrified	115 (11.1)	42 (39.6)	<.001	8.8±25.9	4.9±15.7	.064
Sudden move or loss of home and possessions	81 (7.8)	36 (33.3)	<.001	3.0±11.5	10.9±28.8	.089
Suddenly abandoned by spouse, partner, parent, or family	78 (7.8)	34 (31.5)	<.001	2.6±11.3	10.0±28.1	.057
Did any of these things really bother you emotionally?	434 (42.0)	88 (81.5)	<.001	—	—	—

PTSD, posttraumatic stress disorder.

Data are n (%) or mean±SD unless otherwise specified.

Bold indicates statistical significance (*P*<.05).

* *P* values are from χ^2^ or Mann–Whitney *U* test as appropriate.

**Table 5.** Proportion (Relative to the Total Number of Posttraumatic Stress Disorder Negative and Positive Participants) and Average Frequency of Traumatic Events Reported on the Trauma History Screen Among Civilian and Military Participants Who Screened Positive for Posttraumatic Stress Disorder

Trauma History Screen Question	Proportion			Frequency		
	Civilian Family Members (n=87)	Military Service Members (n=21)	*P* *	Civilian Family Members (n=87)	Military Service Members (n=21)	*P* *
Witnessing a really bad car, boat, train, or airplane accident	28 (32.2)	5 (23.8)	.455	1.5±0.9	1.4±0.5	.962
Witnessing a really bad accident at work or home	7 (8.0)	3 (14.3)	.376	1.7±1.1	35±55.4	.067
Witnessing a hurricane, flood, earthquake, tornado, or fire	26 (29.9)	4 (19.0)	.320	13.9±32.9	1.5±1.0	.505
Hit or kicked hard enough to injure as a child	21 (24.1)	6 (28.6)	.674	31.3±45.1	50.3±53.3	.759
Hit or kicked hard enough to injure as an adult	11 (12.8)	3 (14.3)	.855	21.9±40.7	35.0±55.5	.692
Forced or made to have sexual contact as a child	30 (35.6)	10 (47.6)	.297	24.6±41.6	42.1±49.1	.214
Forced or made to have sexual contact as an adult	16 (18.6)	10 (47.6)	.005	2±1.3	11.7±30.7	.531
Attack with a gun, knife, or weapon	9 (10.9)	3 (14.3)	.619	1.3±0.7	2.3±1.5	.194
During military service, seeing something horrible/being badly scared	3 (3.5)	12 (57.1)	<.001	1±0.00	2.0±0.9	.132
Sudden death of a close family member or friend	49 (56.3)	13 (61.9)	.642	3.8±14.2	1.8±1.2	.826
Seeing someone die suddenly or get badly hurt or killed	24 (27.9)	8 (38.1)	.361	6.0±21.3	2.1±1.0	.059
Some other event that made you feel very scared, helpless, or horrified	32 (37.6)	10 (47.6)	.403	1.9±1.0	13.8±30.8	.450
Sudden move or loss of home and possessions	31 (35.6)	5 (23.8)	.302	8.6±25.6	27.0±48.0	.024
Suddenly abandoned by spouse, partner, parent, or family	28 (32.2)	6 (28.6)	.749	11.8±30.9	1.8±1.2	.581
Did any of these things really bother you emotionally?	70 (80.5)	18 (85.7)	.578	—	—	—

Data are n (%) or mean±SD unless otherwise specified.

* *P* values are from χ^2^ or Mann–Whitney *U* test as appropriate.

**Table 6.** Self-Reported Combat Exposure Scale Scores for Pregnant Military Service Members Who Screened Negative and Those Who Screened Positive for Posttraumatic Stress Disorder (n=298)

Combat Exposure Scale	PTSD Screen Result		*P* ‡
	Negative (n=277)*	Positive (n=21)†	
Light exposure to combat (0–8)	255 (92.1)	13 (70.7)	<.001
Light to moderate exposure to combat (9–16)	13 (4.7)	4 (19.0)	
Moderate exposure to combat (17–24)	3 (1.1)	4 (19.0)	
Moderate to heavy exposure to combat (25–32)	3 (1.1)	0 (0.0)	
Heavy exposure to combat (33–41)	0 (0.0)	0 (0.0)	

PTSD, posttraumatic stress disorder.

Data are n (%) within respective groups who self-reported positive for each variable.

* Posttraumatic Stress Disorder Checklist score lower than 38.

† Posttraumatic Stress Disorder Checklist score 38 or higher.

‡ *P* values are from χ2 test.

## DISCUSSION

In this prospective cohort study, the prevalence of screening positive for PTSD was 9.4%, with similar rates for military (7.0%) and civilian (10.3%, *P*=.101) participants, and was associated with increased risk of depression and at-risk alcohol use (identified with a positive T-ACE screen result at the initial obstetrician visit at or before 16 weeks of gestation) but was not associated with adverse outcomes. This PTSD rate is slightly higher than the 6–8% in a meta-analysis of pregnant civilians^45^ and greater than the PTSD prevalence of 1.7% among pregnant service members in a retrospective study.^15^ Fewer than one in five participants who screened positive for PTSD in pregnancy reported prior diagnosis or treatment for PTSD (Appendix 1, http://links.lww.com/AOG/D986). Although we cannot determine reasons for this low prepregnancy PTSD diagnosis, underdiagnosis is possible. Current recommendations include using validated screening tools for depression or anxiety but do not include screening for PTSD.^46–49^ Routine screening for PTSD in pregnant and postpartum individuals may be beneficial; our study found that the prevalence of PTSD was similar to the rate of perinatal depression (10–14%).^46,50^

There was a significant association between positive PTSD screen results and positive depression screen results at the initial obstetrician visit, with a 17-fold increased risk for positive depression screen results and a fivefold increased risk for positive screen results for at-risk drinking. Although prior studies have reported an increased rate of concurrent PTSD and alcohol use disorder in military veterans,^51,52^ this is one of the first prospective studies to assess PTSD rates among pregnant military-affiliated individuals. Despite the association of positive PTSD screen results with depression and risk for alcohol use, there was no association between positive PTSD screen results and preterm birth, preeclampsia, gestational diabetes, fetal growth restriction, or NICU admissions; however, the low rates of these pregnancy complications likely affected our results. We did find that individuals who screened positive for PTSD had more outpatient visits in pregnancy, which may reflect additional behavioral health visits; however, we cannot confirm the nature of these visits.

The lack of association between positive screen results for PTSD and adverse pregnancy outcomes is consistent with some prior retrospective studies that found no relationship between adverse neonatal outcomes, including preterm birth, small for gestational age, or birth defects, in a pregnant military population with PTSD.^53^ In contrast to other studies showing a relationship between PTSD and adverse pregnancy outcomes,^54^ the rate of complications was low, and our study was underpowered to assess differences in pregnancy complications. Prior retrospective studies found that service members with PTSD initiated prenatal care at earlier gestational ages and had higher prenatal care utilization.^53^ In the current study, there was a significant increase in outpatient visits in individuals who screened positive for PTSD in pregnancy, with five additional visits compared with patients who screened negative for PTSD and military individuals having twice the number of outpatient visits compared with civilians (Table 3).

Among participants who screened positive for PTSD, 50.9% screened positive for depression and 14.8% for at-risk alcohol use. The high rates of historical comorbid mental health conditions support the importance of a thorough mental health assessment and history in individuals who screen positive for PTSD, which may improve identification of individuals at risk for other behavioral health conditions in pregnancy. Proactive identification of individuals who screen positive for PTSD may also improve screening and interventions for alcohol use during pregnancy.

Screening for at-risk alcohol use is important because substance use disorder is associated with a threefold increase in suicide risk in individuals with behavioral health disorders.^55^ Among pregnancy-associated deaths in the United States, 11.4% were attributable to drugs and 5.4% to suicide,^56^ and fetal alcohol spectrum disorders (FASD) affect up to 1 in 20 school-aged children in the United States.^57^ Despite recommendations for abstinence from alcohol in pregnancy, recent studies estimate that 11.5% of pregnant women report alcohol use in pregnancy, and 3.9% report binge drinking in the past 30 days in pregnancy.^58^ Universal screening for alcohol use and brief intervention is recommended, yet FASD remain an underrecognized health inequity,^59^ and we identified a 5.6-fold increased rate of at-risk drinking among individuals with positive PTSD screen results (Table 2). Screening for alcohol use in combination with PTSD screening may improve identification and intervention among individuals at elevated risk for FASD and suicide.

Although one cannot determine causality, the prevalence of positive PTSD screen results was similar in both military service members and civilian family members, which may be attributable to underlying differences in this population. Despite similar rates of PTSD, there were significant differences in reported Trauma History Screen traumas and frequencies between miliary and civilian participants (Table 5). Military individuals with positive PTSD screen results reported a greater proportion and frequency of traumas than civilians. These differences did not translate to differences in pregnancy outcomes or differences in depression and at-risk drinking between military and civilian participants.

The military presents a unique environment, with added stressors and additional military resources. Military-specific stressors are not related solely to combat exposure and include family separations and frequent relocations. In addition, military service members and their families have certain benefits such as covered medical care, stable income, and resources that are not always available in the civilian community. Because our study screened during early pregnancy, identification of participants with presumptive PTSD may have prompted early referral and treatment. It is possible that additional support and access to medical care and insurance in the military health care system mitigated some of the risks of PTSD in pregnancy.

Future studies should investigate interventions that may improve perinatal outcomes associated with PTSD in pregnancy; elevated posttraumatic stress symptoms have been associated with elevated postpartum depression symptoms, physical problems after delivery, and lower health-promoting behaviors.^60^ Social support has also been shown to be a stronger protective factor for PTSD in women compared with men.^61^ Our study found high rates of PTSD in both military and civilian individuals but without associated adverse pregnancy outcomes despite high rates of comorbid behavioral health diagnoses. It is possible that social factors such as early prenatal care and universal health care coverage or support programs such as free, confidential nonmedical counseling offered through Military OneSource (https://militaryonesource.mil/non-medical-counseling/) may have led to improved outcomes, and such factors could be assessed in future studies adequately powered to assess pregnancy outcomes.

The strengths of this study include the large prospective cohort including military service members and civilian family members. These results are likely generalizable to communities with military and veteran populations in the United States. Another strength is that validated screening instruments were used, with the screening cut point for PCL chosen to make presumptive diagnoses of PTSD.

Limitations include the facts that the study was underpowered to detect a difference in preterm labor and depression and that a relatively small number of military service members (n=21) screened positive for PTSD with limited statistical power to generalize nonsignificant differences in pregnancy complications. Additional limitations include voluntary participation, which may have led to selection bias, self-reported behavioral data potentially affected by recall bias, and nonresponse bias. Participants were required to initiate prenatal care early in pregnancy, and it is possible to have missed individuals who would have screened positive for PTSD and may have presented for prenatal care before 16 weeks of gestation, and study results may not be generalizable to individuals presenting later for prenatal care. There is some similarity in the questions included in the PCL and the Edinburgh Postnatal Depression Scale related to interest and enjoyment in activities, which may have increased the probability of a positive score on both screens.

Another limitation is that we do not have information on whether positive PTSD screen results were confirmed with diagnosis and treatment for PTSD. Although information was collected on trauma exposures and a greater number of service members reported having experienced sexual abuse, information was not collected on military sexual trauma. Military sexual trauma is consistently associated with behavioral complications and may be an important contributor to adverse outcomes among pregnant military individuals.^62^ Among pregnant participants who screened positive for PTSD, military service members were more likely to report physical or sexual abuse as an adult. Additional investigation into potential military sexual trauma effects would be important in future studies.

Posttraumatic stress disorder is a significant problem in a pregnant military-affiliated population, affecting approximately 10% of pregnant individuals. Positive PTSD screen results were associated with a 17-fold increased risk of positive depression screen results and a fivefold increased risk of positive screen results for at-risk drinking and increased outpatient visits. Clinicians should be sensitive to possible PTSD in military and veteran-affiliated patients. Strong consideration should be given to including routine PTSD screening in pregnancy and postpartum because it may be at least as common as depression and anxiety.
